# Mr. Thackeray's Case of Death Caused by Poison

**Published:** 1800-10

**Authors:** Frederic Thackeray

**Affiliations:** Emanuel College, Cambridge


### *To the Editors of the Medical and Phyſical Journal.*

Gentlemen,

I Do not remember in your valuable Journal, any diſcuſſion where death was occaſioned by poiſon; if you think the following will aſſiſt your uſeful intentions, its inſertion will much gratify your conſtant reader,

FREDERIC THACKERAY.

*Emanuel College, Cambridge, July* 18, 1800.

The ſubject was a child eighteen months old, in perfect health on the 15th, at eight in the evening: About half paſt eight was ſeized with vomiting and purging, ſucceeded by coma of ſome hours, and died violently convulſed at three in the following morning. As the cauſe was unknown, the child had only taken a little magneſia and rhubarb. I was deſired to examine the body with Mr. R. Criſpin, an able ſurgeon, at Royſton, and opened it thirty hours after its death. The exterior of it exhibited nothing unuſual; no ſwelling nor diſcolouration on any part of it. The veſſels of the inteſtines in various parts, were very full, as if minutely injected. The ſtomach had its external veſſels very much diſtended with blood, dark and very fluid, and a bluſh pervaded every part of its external coat. It was moderately diſtended with air, and about two ounces of a black viſcid fluid, with ſeveral cluſters of mucus or coagulable lymph in it. The inner ſurface of it was very red; and in ſome parts, particularly about the pylorus, it had a lividneſs approaching to mortification, and was ſtudded throughout with a white powder, ſeveral grains of which were matted with ſome mucus, with which ſome experiments were made; and when expoſed to the flame of a candle, fumes and a garlic odour were emitted, proving it was arſenic; of which there can be no doubt, as the girl afterwards confeſſed that ſhe had given arſenic to the infant.

